# Electrochemotherapy in the Treatment of Head and Neck Cancer: Current Conditions and Future Directions

**DOI:** 10.3390/cancers13061418

**Published:** 2021-03-19

**Authors:** Tomohiro Enokida, Makoto Tahara

**Affiliations:** Department of Head and Neck Medical Oncology, National Cancer Center Hospital East, 6-5-1 Kashiwanoha, Kashiwa 277-8577, Japan; tenokida@east.ncc.go.jp

**Keywords:** electrochemotherapy, cisplatin, bleomycin, head and neck cancer, immunotherapy, quality of life

## Abstract

**Simple Summary:**

Electrochemotherapy (ECT) was first introduced in the late 1980s and was initially used mainly on cutaneous tumors. It has now evolved into a clinically verified treatment approach. Thanks to its high feasibility, it has been extended to treating mucosal and deep-seated tumors, including head and neck cancer (HNC) and in heavily pretreated settings. This review describes current knowledge and data on the use of ECT in various forms of HNCs across different clinical settings, with attention to future clinical and research perspectives.

**Abstract:**

Despite recent advances in the development of chemotherapeutic drug, treatment for advanced cancer of the head and neck cancer (HNC) is still challenging. Options are limited by multiple factors, such as a prior history of irradiation to the tumor site as well as functional limitations. Against this background, electrochemotherapy (ECT) is a new modality which combines administration of an antineoplastic agent with locally applied electric pulses. These pulses allow the chemotherapeutic drug to penetrate the intracellular space of the tumor cells and thereby increase its cytotoxicity. ECT has shown encouraging efficacy and a tolerable safety profile in many clinical studies, including in heavily pre-treated HNC patients, and is considered a promising strategy. Efforts to improve its efficacy and broaden its application are now ongoing. Moreover, the combination of ECT with recently developed novel therapies, including immunotherapy, represented by immune checkpoint inhibitor (ICI)s, has attracted attention for its potent theoretical rationale. More extensive, well-organized clinical studies and timely updating of consensus guidelines will bring this hopeful treatment to HNC patients under challenging situations.

## 1. Introduction

Head and neck cancer (HNC) accounts for more than 5% of all malignancies worldwide. Around 90% of cases are squamous cell carcinoma (SCC) [1]. Approximately two-thirds of patients with HNC present with advanced-stage disease and are primarily treated with both surgical resection and irradiation as curatively intended treatments [2]. Despite aggressive, site-specific multimodality therapy, however, a significant proportion of patients will develop disease recurrence, with up to 60% risk of local failure and 30% of distant failure [3,4]. Besides, several malignancies other than HNC can occur in the head and neck region, including melanoma and cutaneous carcinoma, represented by basal cell carcinoma (BCC). For these, surgery remains the mainstay of treatment. Moreover, second primary tumors frequently develop in these patients, at a rate of 2–3% per year. Treatment of recurrent and second primary tumors is particularly challenging, especially when radiotherapy is no longer an option because of previous irradiation or when the site has already been subjected to extensive surgery, mainly due to concerns about toxicity and effect on the quality of life (QOL), loss of function and cosmetic disfigurement [5,6]. In fact, only a few patients with locoregional recurrence can be salvaged by surgery or reirradiation. If all curative treatment options are exhausted, the patient eventually becomes a candidate for palliation, which is provided by the administration of various systemic chemotherapy regimens. Prognosis in these cases is unfortunately still low, with a median overall survival (OS) of 10–15 months [7]. For these reasons, new treatment modalities that can control local growth and diminish local symptoms are of critical interest. Among these, electrochemotherapy (ECT) has shown promising efficacy, a low frequency of side effects and an organ--sparing effect, which together result in minimal function impairment, as demonstrated in multicenter studies [8,9]. The purpose of this review is to clarify current knowledge and existing data on the use of electrochemotherapy for HNC, with attention to future clinical and research perspectives.

## 2. Electrochemotherapy (ECT)

### 2.1. Mechanism

**Figure 1:**
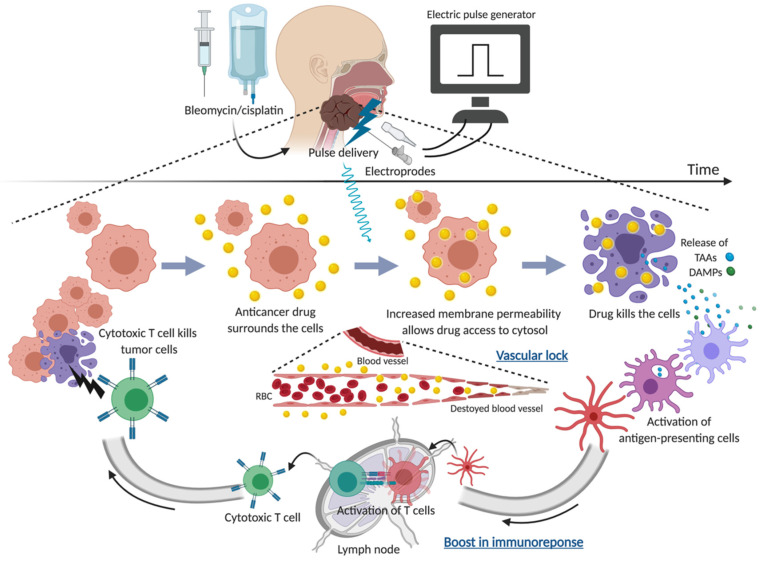
The concept of electrochemotherapy in HNC. TAAs, tumor-associated antigens; DAMPs, damage-associated molecular pathways.

The basic concept of ECT is shown in Figure 1. ECT was first introduced in the late 1980s and has now evolved into a clinically verified treatment approach for cutaneous and subcutaneous tumors. Its use has recently been extended to deep-seated tumors [10,11,12,13,14]. Mechanistically, this local cancer treatment modality combines local or systemic administration of a chemotherapeutic drug, for example cisplatin and bleomycin, with temporal permeabilization with locally applied short-intensity high-voltage pulsed electric pulses to tumor cells. Once the electric pulses have exceeded the cell’s membrane potential, a depolarization process occurs whereby the cell membrane becomes temporarily permeable, allowing the transportation of typically poorly penetrating chemotherapeutic drugs from the extracellular into the intracellular space of tumor cells. It results in an increased amount of drug in the cell; eventually a higher cytotoxic effect is obtained [15,16,17,18,19,20,21]. Unlike alternative ablative therapies, electroporation does not affect the structural integrity of the surrounding tissue, thereby enabling the treatment of tumors in the vicinity of vital structures. This technique has also led to new research into DNA vaccine delivery and gene therapy [22,23]. In addition to the direct antitumor function of the drug, ECT has several mechanisms of action, which may involve vascular effects and an immune response. The former, a combination of the drugs and the electric pulses, causes vasoconstriction and endothelial cell death in afferent tumor vessels and subsequent blockage of tumor blood flow [24,25]. This vascular disruption leads to drug entrapment (“vascular lock”) and tumor ischemia, which consequently contributes to highly efficient antitumor effects as well as reduced bleeding when invasive electrodes (i.e., needles) are used - which is particularly advantageous in well-vascularized tumors [26,27]. The latter results from tumor cell destruction during ECT. This can induce the recruitment of antigen-presenting cells (APC) from peripheral blood through tumor-associated antigen exposure as well as the release of damage-associated molecular patterns (DAMPs), such as heat shock proteins and calreticulin, which are secreted from cancer cells in response to ECT. The DAMPs altogether confer a robust adjuvanticity to dying cancer cells, as they favor the recruitment and activation of antigen-presenting cells (immunogenic cell death (ICD)). This eventually works like an in situ cancer vaccine [28,29]. The preclinical studies highlighted that ECT-mediated tumor regression was dramatically decreased in animals depleted of functional T lymphocytes, in comparison to immunocompetent mice [30,31,32]. Moreover, data representative of these immunologic mechanisms of ECT have been reported in clinical studies involving patients affected by advanced melanoma [33,34,35,36].

### 2.2. Procedures

Technically, the first step is administration of the chemotherapeutic drug. The most commonly used is bleomycin, owing to its limited transport through the plasma membrane, high specificity for tumor cells without substantial damage to surrounding tissue, and the availability of intratumoral administration, which provides equal treatment efficacy with that of intravenous administration [8,37,38,39,40,41]. Once inside the cell, bleomycin causes single- and double-strand DNA breaks leading to quick cell death by pseudoapoptosis [42].

For example, in preclinical studies, ECT potentiated the cytotoxicity of bleomycin 300- to 700-fold and that of cisplatin up to 12–70-fold [16,43,44,45,46]. Clinically, bleomycin with electroporation produced a significantly greater number of patients with partial or complete response to therapy than that with bleomycin alone in patients with advanced squamous cell carcinoma of the head and neck (SCCHN) (57% vs. 3%: *p* < 0.001) [47]. ECT with intralesional cisplatin might be a valuable alternative to ECT with bleomycin without deputizing efficacy in those with renal disease, in the elderly, and in all who might experience severe adverse effects with this drug [48]. However, the comparatively limited increase in efficacy of ECT with intravenous cisplatin compared with intravenous cisplatin alone, especially in head and neck metastases, has limited broader clinical interest [44]. These findings for intralesional cisplatin would likely be beneficial to recurrent HNC patients with impaired renal function due to prior anti-cancer treatment. A meta-analysis confirmed that ECT had significantly higher effectiveness than bleomycin and cisplatin alone [8,47]. Furthermore, a systematic review on ECT in the management of primary and metastatic cutaneous malignant tumors indicated similar anti-tumor efficacy between the two drugs (overall response (OR) rate, defined by complete response (CR) plus partial response (PR)), for bleomycin and cisplatin was 83.9% and 80.8%, respectively) [49]. On the other hand, using recent cell lines and tumor models treated with ECT, Prevc et al. showed that HPV―positive pharyngeal SCC responds better to ECT with cisplatin than HPV―negative tumors, and also responds better than to ECT with bleomycin [41].

The chemotherapeutic drug is administered intratumorally (bleomycin or cisplatin) or intravenously (bleomycin) or, rarely, intraarterially, depending on the number and size of tumors, as well as on patient features like pulmonary and renal function [50,51]. Intratumoral administration of bleomycin requires a smaller dose than intravenous administration, and larger-volume tumors are generally thought to be more readily treated by intravenous than intratumoral administration. Larkin et al. proposed intratumoral administration of bleomycin when the patient has few nodules of less than 3 cm in maximal diameter; otherwise, they prefer an intravenous route [46]. Gehl and Sersa et al. also suggested considering debulking the exophytic element within the electrochemotherapy session in large tumors (exophytic more than 3 cm thick) in their standard operating procedures for cutaneous tumors and skin metastases [51]. Besides, intratumoral administration may provide more efficacious treatment for poorly-vascularized tumors [44,52,53,54,55]. Doses for intratumoral injection are determined by tumor volume. An example dose might be ~500 IU/cm^3^ bleomycin or 1 mg/cm^3^ cisplatin [55]. Tumor volume is usually calculated by imaging. Allegretti et al. measured tumor volumes with the formula for an ellipse, V = abc π/6, and this has been widely adopted [56]. In contrast, the dose for intravenous administration of bleomycin is based on body surface area (in square meters; 15,000 IU/m^2^ bleomycin). Following administration, electrodes are either inserted into or push against the tumor. For systemic delivery, pulses require administration during the pharmacokinetic peak for maximum treatment efficacy, which is between 8–28 min following drug administration versus 1–10 min with intratumoral delivery [52,53,54]. There are three types of fixed geometry electrodes, such as hexagonal and pentagonal configurations with a central electrode in each, plate electrodes, and needle electrodes. Effective ECT requires that the electrodes are applied so as to ensure complete coverage of the entire tumor and these are thus used differently depending on the case: treatment of superficial lesions, such as those of the skin, is typically performed with plate electrodes, whereas long single-needle electrodes allow for ECT of deep-seated tumors of the head and neck, including the oral and nasal cavity and pharyngeal-laryngeal lumen [10]. Ultra-short electric pulses are delivered; typically, square wave electric pulses of ~100 µs with a field strength of 1300 V/cm [44,57], grouped in runs of 4, 6, or 8 over a 40-min period. Small tumors may be treated with a single run while larger tumors require moving the electrodes step-by-step according to the permeabilization coefficient for EP of the whole target area [57]. Application of ECT is usually followed by inflammation, with differing amounts of tissue swelling and tumor necrosis.

In the head and neck, a single ECT treatment may be sufficient for single or multiple tumor nodules, including head and neck metastases [58]. This treatment may completely eradicate tumor nodules. Less dramatically effective treatment can be repeated as often as monthly (with an interval of at least four weeks). Larger tumors >3 cm in size can be successfully treated by repetitive application of electric pulses to the tumor until the whole tumor area is covered [58]. Intraoperative anesthetic management depends on disease extent and anatomic location along with electrode type. Local anesthesia may be sufficient for treatment of smaller tumors, such as skin metastases; however, general anesthesia is best suited when deep-seated or open surgery is indicated, as well as with superficial tumors of the face, scalp, oropharynx and other sensitive areas to ensure patient comfort and maintain airway control [8,58,59]. ECT represents a less invasive approach, but post-treatment tissue swelling may require elective tracheostomy when ECT was performed in oral cavity or oropharyngeal tumors. For details on eligibility criteria, dosage and route of bleomycin/cisplatin, choice of electrodes, type of anesthesia, follow-up period, and specific operating procedures we suggest the European standard operating procedures (SOP) in the Electrochemotherapy (ESOPE) guidelines [50]. Moreover, the SOP for ECT of cutaneous tumors and skin metastases has recently been updated [60]. New recommendations based on expanded experience and treatment of larger tumors in a larger and more diverse patient population have also appeared [51,61]. In addition, the National Institute for Health and Care Excellence (NICE, UK, www.nice.org.uk, accessed on 1 November 2020) has issued guidelines for electrochemotherapy [62,63].

## 3. Current Clinical Application of ECT

The first clinical trial using electrochemotherapy with bleomycin was performed in France in 1991 by Mir et al. [64]. Since then, electrochemotherapy has undergone numerous trials on advanced malignant melanoma (MM), [26,58,59] and non-melanoma skin cancer, [50,59,65] cutaneous recurrent breast cancer, [66] superficial soft tissue sarcoma, tumors in the cervicofacial region, some gynecological tumors, [67] as well as chronic lymphocytic leukemia infiltration [68]. According to a report from ESOPE involving various forms of cancer, including cutaneous and subcutaneous melanoma nodules, in 2006, OR rate was 85% and a favorable local tumor control rate at 150 days after treatment was achieved across the different approaches (88% with bleomycin administered intravenously, 73% with bleomycin administered intratumorally and 75% with cisplatin administered intratumorally) [50]. Systematic reviews of ECT in the management of primary and metastatic cutaneous malignant tumors showed OR rates of 82.2–84.1% and CR rates of 46.6–59.4% [8,49,69]. Interestingly, objective response increased significantly after publication of the ESOPE results: on comparison of CR and OR rates in studies published before and after the ESOPE study, the difference in CR rates was not significant whereas that for OR was (*p* = 0.565 for CR, *p* < 0.001 for OR) [8]. The International Network on Sharing Practices on Electrochemotherapy (InspECT) reported an overall response rate in 74% of 114 patients with metastatic malignant melanoma and 78% of 394 lesions treated by ECT using intravenous or intratumoral injection of bleomycin [70]. Today, a wider range of subjects are eligible for treatment, and research into use in colorectal cancer [71], hepatocellular carcinoma [14], and liver metastases [13,72] is ongoing.

### 3.1. Efficacy of ECT in HNC

Table 1 presents the principal findings of ECT treatment in HNC [47,48,59,65,73,74,75,76,77,78,79,80,81,82,83,84,85,86,87,88,89,90,91,92]. Although the literature describing HNC do not always make clear whether the cancer location is cutaneous or mucosal, this review will focus on both cutaneous tumors and mucosal tumors (located in, or derived from, a mucosal surface, such as in the oral cavity, pharynx, larynx) in the head and neck region. To date, most studies on efficacy have been based on relatively small case studies. Patients with advanced inoperable skin tumors are frequently left with only a few therapeutic options. Experience with ECT in the head and neck area has been mainly obtained with primary or metastatic skin cancers, the majority cutaneous nodules or subcutaneous tumors [88]. One study of ECT with bleomycin for cutaneous metastases, including in the head and neck area, showed a CR of 68% and PR of 18% [65]. BCC of the skin is usually found in the head and neck region, especially in areas such as the pinna, outer ear in general, and nasal tip. In 2016, Rotunno et al. reported a CR rate of 75% in patients with head and neck BCC treated by ECT. The majority (42%) of these tumors, which had a median size of 24 mm, were located on the scalp and half of the patients required at least two ECT cycles [81]. The EURECA group has reported promising results with ECT in patients affected by recurrent, metastatic, or primary skin cancer of the head and neck area not suitable for surgery or chemo/radiotherapy across the histological type of the tumor: after one year of follow-up, overall disease-free survival (DFS) was 89%, broken down as 87% for SCC, 100% for BCC and 89% for malignant melanoma [59]. In addition, cutaneous angiosarcomas, which are more common in the head and neck region and account for more than 60% of cases [93], has also been treated with ECT. A multicenter retrospective analysis which reviewed the cases of 19 patients (five with scalp angiosarcoma) who underwent ECT for superficial advanced angiosarcomas reported an OR rate of 63% at 2 months after treatment and a 6-month disease stabilization rate of 47% [82]. In a systematic review, Lenzi et al. included 16 studies on both skin and mucosal SCCHN and reported the data of 200 treated patients [91]. The combined results show a very heterogeneous OR rate, ranging from 0% to 100% (in 14 of the 16 studies, overall response rate was higher than 50%), while CR rate ranged between 0% and 83.3% [94]. Possible explanations for this include heterogeneity in the primary site, prior treatment, tumor size, and concomitant (e.g., neck dissection) or post-ECT treatment (e.g., post-ECT chemoradiotherapy) [94]. On the other hand, Longo et al. evaluated palliative ECT treatment in a heterogeneous group of patients with either or both recurrent and metastatic head and neck cutaneous or mucosal tumors, consisting mostly of SCCHN, who had chemo- and radio-refractory disease not suitable for surgery and at least two previous chemotherapy lines in addition to radiation therapy [89].

The OR rate was 45%, with 5% CR, and median OS time was 9.1 months, which is promising given that the median overall post-failure survival of patients with inoperable loco-regional failure in SCCHN after radical radiotherapy was 7.4 months, even without a prior history of systemic chemotherapy [95]. Furthermore, ECT was prospectively tested in the palliative setting in patients with HNC, including those with a mucosal, cutaneous and salivary gland origin who already been treated with either or both surgery and chemoradiotherapy and had absolutely no other therapeutic option [90]. While 1-year OS rate and median OS under best supportive care in these cases are up to approximately 15% and 5 months, respectively [90,91,96,97] the reported OS probability at 1 year in this study was 41.6% (median OS: 9 months), suggesting encouraging efficacy in this challenging population [90].

Several studies have featured mucosal HNC. Plaschke et al. performed a systematic review on ECT of mucosal head and neck tumors [91]. As of February 2016, they found only 11 studies with a total of 72 patients with transmucosal ECT treatment, including 36 patients with primary tumors, and reported a good overall response to ECT [98]. In a prospective trial of six European institutions (European Research on Electrochemotherapy in Head and Neck Cancer–EURECA) reported in 2017, ECT was investigated in 37 patients with recurrent and mucosal head and neck tumors in whom standard treatments had either failed, were not deemed suitable or were declined by the patient. An OR of 56% (CR rate: 19%) with a 1-year OS rate of 54% was observed, and three patients (7%) remained at CR at 30, 34, and 84 months post-treatment [84]. The Copenhagen group doubled their series of patients included in the EURECA trial to 26 patients with recurrent mucosal HNC and no curative treatment option and reported a similar OR rate of 58% (CR rate 19%) measured eight weeks after treatment [91]. Furthermore, moves to focus on certain subsites of mucosal HNC have been made. Gargiulo et al. reported the effectiveness of a single session of ECT with bleomycin in 21 patients with primary or recurrent lower lip SCC as a specific subsite, including three patients treated in the palliative setting and four patients who received surgery following ECT. The OR rate following a single session of ECT was 100%, with CR in 71.4% and PR in 28.6% of patients [87].

Across various study types, factors which predict treatment outcome in the field of HNC treated with ECT have been shown. It is generally accepted that a better response to ECT is obtained with small lesions (e.g., ≤3 cm in diameter in the skin and or mucosal HNC [59,76,89], or stage T1/2 in mucosal HNC [98]). Specifically, tumors ≤3 cm in diameter showed an OR rate of 88% versus 68% in those >3 cm [59]. Possible reasons for the better response in smaller lesions include insufficient exposure to the chemotherapeutic drug due to inadequate blood flow and higher interstitial pressure in larger tumors, and insufficient coverage of the tumor by the electric field due to difficulty in applying the electrodes to larger tumors [69]. Further, treatment-naïve tumors responded significantly better than previously treated tumors (*p* = 0.0269) [59]. Interestingly, for recurrent tumor nodules, previous surgery least affected the possibility of achieving CR compared to a history of chemotherapy or radiotherapy [59], possibly due to the selection of highly resistant clones during previous treatment [67]. Response to ECT in previously irradiated fields could be limited by partial electrode needle penetration and suboptimal electrical current delivery in fibrotic tissue, as well as the possible selection of highly resistant clones during previous treatment [49,67]. These findings are compatible with those in the studies which included other tumor sites [65,67,70].

### 3.2. Safety of ECT in HNC

Because of its mechanism of action, ECT is able to reduce damage to healthy tissue, leading to a limited side effect profile. As a general consensus, small--size primary cancers without previous treatment are more likely to show a good response, resulting in lasting tumor control, fewer side effects, and better clinical response with less bleeding, pain, and erythema, whereas large recurrent tumors display more frequent side effects, including treatment-related pain and some serious adverse events [48,91]. The most common toxicity is post-operative pain, which reaches a peak at week 3–4, then usually decreases during the healing phase depending on tumor control [80]. Several studies reported that tumor size, previous irradiation, and a high pain score before ECT were predictors of increased pain after ECT [99]. In this context, repeated treatment of lesions on the scalp has been associated with poor spontaneous healing rates [59,84]. Other minor side effects of ECT in the HNC region include hyperpigmentation, maculopapular rash, odor, and headaches [84]. The prospective EURECA study in 105 skin HNC patients treated with ECT reported one major adverse event, a patient with a large ulcerated tumor who died due to septic shock on the second day after the procedure [59]. The other ECT-related toxicity that should be mentioned in HNC is post-treatment bleeding. There are two reported cases of bleeding from the parapharyngeal area requiring admission for monitoring, one from among 44 patients with SCCHN of the base of the tongue and the second from 19 primary mucosal HNC patients treated by ECT [47,80]. In contrast, two cases with internal carotid artery (ICA) involvement and a history of external beam radiotherapy are notable for their successful partial or complete response to treatment; neither patient experienced hemorrhagic or neurologic complications, likely due to careful pre-treatment procedures such as balloon test occlusion of the ICA [56]. Although we should note that these are unadjusted non-comparative descriptive data from a small number of patients and require confirmation in larger studies, the safety profile of ECT in this population is encouraging, particularly given the relatively high incidence of carotid blow-out (2.6–4.1%) reported in re-irradiation studies for recurrent or second primary HNC [100,101,102]. Further, as ECT treatment causes necrosis, post-treatment refractory fistula has been seen, mostly in cases with extensive full-thickness lesions (e.g., cheek or floor of mouth). These potentially raise the risk for secondary bleeding and may require additional procedures, such as feeding tubes or percutaneous endoscopic gastrostomy (PEG) [59]. As with post-treatment bleeding, it is difficult to conclusively establish if the cause of these events was locoregional progression or local deficit by the treatment effect; in either case, this possibility adds to the list of reasons for careful patient selection. Although the cumulative doses of bleomycin used in ECT are much lower than those routinely used in testicular carcinoma, for example, the possibility of bleomycin-induced pulmonary fibrosis has also been raised [84]. The overall incidence of grade ≥3 toxicity in studies of HNC treated with ECT was 5.7–11% [59,84], which is generally lower than those seen with systemic therapy, the current standard of the care (e.g., 55–85% in KEYNOTE048 study of anti-PD-1 ab pembrolizumab +/− chemotherapy as 1st line treatment for r/m SCCHN) [7].

This favorable efficacy and safety profile of ECT will contribute to improving patient QOL, which is a vital treatment outcome in patients who harbor tumors in the head and neck region, in whom functional (e.g., voice impairment, difficulty in swallowing), physical (e.g., treatment-related mucositis, osteoradionecrosis) and psychosocial (e.g., changed cosmetic appearance) problems can persist due to the disease itself and conventional treatment procedures.

### 3.3. Effect on QOL of ECT in HNC

To date, the impact of treatment on the individual’s QOL in studies for recurrent or metastatic disease has primarily been evaluated using a unified questionnaire, such as the European Organization for Research and Treatment of Cancer core 30 quality-of-life questionnaire (EORTC QLQ-C30), the EORTC 35-question head and neck cancer-specific module (QLQ-H&N35), and the EuroQoL 5-dimensions questionnaire (EQ-5D) [103,104]. Analysis of QOL in patients with skin cancer of the head and neck area showed a significant progressive, positive perception of well-being using the EQ-5D and a significant improvement in physical functioning, role functioning, and decrease in fatigue and pain with the QLQ-C30. Further, there was a general improvement in all domains with the QLQ-H&N35, with the most significant improvements seen in perception of feeling ill, pain and use of analgesics, and mouth opening [59]. These questionnaires also worked in identifying significantly improved well-being, social eating, and swallowing in mucosal HNC patients treated with ECT [84]. As a simpler method to comprehensively assess treatment impact on the patient’s subjective pain, the visual analog scale (VAS) [105], which is a validated, subjective measure for acute and chronic pain (scores are recorded by making a handwritten mark on a 10-cm line that represents a continuum between “no pain” and “worst pain”) has frequently been used [48,90]. ECT for cutaneous and mucosal HNC resulted in significant pain reduction after ECT, presumably because of tumor control; the mean VAS score significantly reduced from 6.08 before starting treatment to 1.25 at one month after ECT (*p* < 0.001) [90]. This finding was clearly reproduced in a second study in which median VAS score before treatment was 6.02 vs. 2 at 1 month after ECT (*p* < 0.001) [89]. In addition to this, further actual patient benefit was reported from other perspectives, including a decrease in hospital visits for local management (dressing) and the control of bleeding: an average of 6.8 hospital visits per month before treatment vs. 1.29 after treatment (*p* < 0.001) [90] and a significant decrease in moderate or severe bleeding from diagnosis after ECT (*p* = 0.00012) [89].

## 4. Future Direction of ECT in HNC

Despite this steady increase in the number of published reports, several factors may be setting back systematic usage and universal availability. These include the heterogeneity of subjects in terms of clinical features (e.g., enrolling both mucosal and skin carcinomas, different histology, clinical stages, and different treatment settings in the same study). In addition, the use of unified measures is required to compare toxicity, QOL and tumor response following ECT across studies. For example, too early evaluation after ECT in a mucosal tumor may not deliver accurate results due to the effect of inflammation and wound healing, as is similarly observed in the post-radiation setting [80,106]. A meta-analysis on 47 prospective studies of 4313 cutaneous metastases treated by any of five skin-directed therapies (ECT, radiation, photodynamic, intralesional, and topical therapies) showed the same, or even superior, effectiveness of electrochemotherapy over other therapies [9]. Regrettably, however, the lack of a control arm with the current standard of care, and of specific data from the homogenous population of interest, including patients harboring cancer in the head and neck region, prevent the drawing of any definitive conclusions in that population. These difficulties point to the desirability of prospective studies which target homogenous populations having standardized and widely accepted treatment protocols, evaluation measures, and control arms of standard care cohorts. Interestingly, although ECT as palliative treatment is a well-established option for patients in an advanced stage of illness, the curative potential of ECT in early-stage (i.e., primary) cancer treatment has yet to be investigated. Recently, 5-year follow-up results of the first non-inferiority prospective randomized control trial evaluating ECT against the gold standard of treatment―surgery―for patients with primary BCC showed statistical equivalence between the two arms, represented by a local disease-free progression of 87.5% with ECT and 97.5% with surgery (*p* = 0.33) [107].

There have been many efforts to further improve the efficacy of ECT and provide a safer toxicity profile. In recent years, immunotherapy centered on anti-PD-1 therapy with or without chemotherapy has come to represent an option for standard of care treatment for all solid tumors, including recurrent or metastatic HNC [7,108]. Nevertheless, an as-yet uncertain percentage of patients will not achieve a clinical response (i.e., OR of nivolumab monotherapy: 13.3% [108]), and some experience rapid tumor progression during treatment (29% [109]), which is particularly problematic in symptomatic patients, most commonly with pain or bleeding, with a bulky lesion on the head or neck. In this context, the combination of ECT plus an immune checkpoint inhibitor (ICI) (electroimmunotherapy) is a promising approach, as ECT can boost the tumor’s immunogenicity as described above and favorably alter the tumor microenvironment by promoting the influx of T cells, potentially leading to improved ICI efficacy [110]. In fact, several preclinical models demonstrated that ECT combined with immunotherapy enhanced local antitumor effects and achieved systemic effects [111,112,113]. Clinically, despite the lack of prospective data with a control arm, electroimmunotherapy (ECT plus either anti-CTLA-4 or anti-PD-1) has shown favorable efficacy without apparently increasing toxicities in several advanced melanoma cohorts [33,34,35,36,110]. We believe that the augmented anti-tumor efficacy of this combination will surely be beneficial to patients with HNC. Other attempts include optimizing the type and dose of administrated drugs during the procedure as well as the concurrent use of ECT with radiotherapy. Regarding the former, Groselj et al. [86] and Jamsek et al. [92] showed that a reduced bleomycin dose (10,000 IU/m^2^) was comparably effective to the standard dose (15,000 IU/m^2^) in elderly (>65 years) patients with nonmelanoma HNC. These findings are beneficial for elderly or heavily pretreated HNC patients who frequently have impaired renal function and decreases in lean body mass and total body water, and at consequent risk of unexpectedly elevated plasma or serum levels of administrated drugs. In addition, the use of intratumorally injected supraphysiological doses of calcium in combination with ECT was recently introduced, and showed similar efficacy with relatively less toxicity than bleomycin in a randomized phase II study [114]. Preclinical studies in murine tumor models with this latter approach have indicated that ECT has a radiosensitizing effect, and that this is moreover retained at low bleomycin dosages [115,116]. Furthermore, Raeisi et al. also demonstrated that concurrent irradiation increases the antitumor effectiveness of ECT with cisplatin on large invasive ductal carcinoma tumors, suggesting that this three-modalities combined treatment is promising [117]. Apart from that, there are many attempts to improve or optimize ECT. Based on the characteristics of oxaliplatin, which are similar to those of cisplatin, and the presumably more pronounced immunomodulatory effect of oxaliplatin, oxaliplatin is a candidate for ECT [118]. As an adjuvant procedure, intramuscular interleukin-12 (IL-12) gene electro-transfer, which provides systemic shedding of IL-12, increased the cure rate in a dose-dependent manner when was combined with local ECT with cisplatin [119]. Besides, the recent report showed that gene electrotransfer of plasmid encoding shRNA against tumor component, herein, melanoma cell adhesion molecule (pMCAM) elicited anti-tumor dual-action including a vascular-targeted effect mediated by silencing of MCAM and an immunological effect mediated by the presence of plasmid DNA in the cytosol activating DNA sensors [21]. Further improvements of ECT by these potentially applicable novel findings are highly anticipated.

Timely updating of the standard operating procedure to reflect these recent novel findings will enrich the significance of this treatment, and we look forward to this as a matter of urgency.

## 5. Conclusions

Many clinical studies indicate that ECT as one component in the treatment of HNC provides encouraging efficacy with an acceptable toxicity profile, even in subjects who are heavily pretreated and have no further treatment options. Combination with other recent advanced therapeutic strategies, including immunotherapy, will further increase its value and treatment opportunities in this challenging population. Larger, well-organized studies in homogeneous subjects and timely updating of consensus guidelines are therefore warranted.

## Figures and Tables

**Table 1:** The studies of Electrochemotherapy in Head and Neck Cancers.

Author	Year	Type of Trial	*N*	Pathology: # of Patients	Drug	Rout *	Outcome *	Note	Ref.
Panje WR et al.	1998	Phase I/II	10	SCC: 8 ACC: 1 ADC: 1	Bleomycin	I.T	OR: 80%(CR:40%)	Two patients who refused surgery and RT were enrolled.	[73]
Hofmann GA et al.	1999	Phase I/II	10	SCC: 8 ADC: 2	Bleomycin	I.T	OR: 80% (SCC: 75%(CR:25%)) (ACC: 100%(CR:50%))		[74]
Rabussay DP et al.	2002	Phase II	54 (North American I study: 17) (North American II study: 25) (European study: 12)	SCC: 54	Bleomycin	I.T	North American I study: 55% (CR: 30%) North American II study: 58% (CR: 19%) European study: 56% (28%)		[75]
Burian M et al.	2003	Prospective	12	SCC: 12	Bleomycin	I.T	OR: 100% (CR: 83.3%)	10 patients received ND concurrent with ECT. Seven patients received RT following ECT.	[76]
Bloom DC et al.	2005	Phase II	54	SCC: 54	Bleomycin	I.T		Two patients withdrew due to adverse events. Eight other patients were treated with BM alone ECT had significantly higher effectiveness (by more than 50%) than bleomycin and cisplatin alone.	[47]
Matthiessen LW et al.	2011	Phase II	51	MM ADC BCC SCC Others	Bleomycin	I.V: 30 I.T: 21	OR: 79%(CR:60%) (Size ≤ 3 cm: 86%(CR:68%)) (Size >3 cm: 31% (CR: 8%))	30 lesions in the head and neck were treated in this study.	[65]
Gargiulo M et al.	2012	Retrospective	25	SCC: 13 (recurrent: 3) BCC: 9 (recurrent: 0) ADC: 2 (recurrent: 2) Bowen: 1 (recurrent: 1)	Bleomycin	I.V	OR: 100%(CR:72%) (SCC:100%(CR:65%) (BCC:100%(CR:100%))	Four patients received surgery following ECT.	[77]
Mevio N et al.	2012	Retrospective	15	SCC: 13 (recurrent: 11) BCC: 1 (recurrent: 1) Merkel cell carcinoma: 1 (recurrent: 1)	Bleomycin	I.V	OR: 94% (CR:61.5%)	Tumor response was evaluated according to the assessable lesion (*n* = 31).	[78]
Campana LG et al.	2014	Retrospective	39	SCC: 24 BCC: 9 ADC: 5	Bleomycin	I.V:7 I.T:7 I.V+I.T:25	OR: 59% (CR:38%) 1-year overall LPFS: 59%	Site of tumor, Skin of the head and neck: 27 Oral cavity and oropharynx: 12 15 patients were treatment-naïve	[79]
Landström FJ et al.	2015	Prospective	19	SCC: 18 AD: 1	Bleomycin	I.T	OR: 100% (CR:100%) 5-year local control in 12 surviving patients: 100% 5-year tumor-specific survival: 75%	All patients had mucosal primary tumor (oral cavity or oropharynx). Two patients received ND concurrent with ECT. 12 patients received RT following ECT.	[80]
Rotunno R et al.	2016	Phase II	55		Bleomycin	I.V	OR: 91% (CR:60%) [BCC in head and neck (CR:75%)]		[81]
Bertino G et al.	2016	Prospective	105	SCC: 50 BCC: 34 MM: 10 Others: 11	Bleomycin	I.V: 97 I.T: 8	OR: 100% (CR:72%) (SCC:79% (CR:55%) (BCC:97% (CR:91%)) (MM:77% (CR:55%)) (Others: 44% (CR:0%)) 1-year overall DFS: 89% (SCC: 87%) (BCC: 100%) (MM: 89%)	This study focused on skin HNCs. 28 patients were treatment-naïve Tumor response was evaluated according to the assessable lesion.	[59]
Guida M et al.	2016	Retrospective	19	Angiosarcoma: 19	Bleomycin	I.V	OR: 63% (CR:42%) 1-year DFS: 68% 1-year PFS: 45%	This study focused on superficial angiosarcomas Five of 19 patients had scalp angiosarcoma	[82]
Di Monta G et al.	2017	Retrospective	22	SCC:22	Bleomycin	I.V	OR: 81.8% (CR:22.7%)	18 of 22 patients had skin cancer of the head and neck. Seven patients received ECT ≥ 2 times.	[83]
Plaschke CC et al.	2017	Phase II	43	SCC:39 ACC: 3 ADC: 1	Bleomycin	I.V: 2 I.T: 41	OR: 56% (CR:19%) 1-year OS: 54% 1-year LPFS: 54%	This study focused on mucosal HNCs. 37 of 43 patients were evaluable for tumor response.	[84]
Montuori M et al.	2018	Prospective	15	BCC SCC	Bleomycin	I.V	OR: 100% (CR:100%)		[85]
Groselj A et al.	2018	Prospective	28	BCC: 42 lesions SCC: 10 lesions [Standard dose: 12 patients] BCC: 17 lesions SCC: 7 lesions (Reduced dose: 16 patients) BCC: 25 lesions SCC: 3 lesions	Bleomycin	I.V (standard dose):12 I.V (reduced dose):16	OR: Standard dose: 100% (CR:100%) (BCC:100%(CR:100%)) (SCC:100%(CR:100%) Reduced dose: 100% (CR:94%) (BCC:100%(CR:96%)) (SCC:100%(CR:100%)	Patients with a primary recurrent skin cancer of the head and neck aged > 65 years old were enrolled. This study compared standard I.V dose bleomycin (15,000 IU/m^2^) and reduced dose (10, 000 IU/m^2^). Tumor response by histological type was evaluated according to the assessable lesion.	[86]
Gargiulo M et al.	2018	Retrospective	21	SCC (lower lip): 21	Bleomycin	I.V	OR: 100% (CR: 71.4%)		[87]
Campana LG et al.	2019	Prospective	20	Angiosarcoma: 20	Bleomycin	I.T	OR: 80% (CR:40%) Median OS: 12.5 months Median LPFS: 10.9 months	This study focused on advanced cutaneous angiosarcomas. Seven of 20 patients had scalp/facial angiosarcoma. Combined with surgery in five patients. Concomitant systemic treatment in three patients.	[88]
Longo F et al.	2019	Retrospective	93	SCC BCC Others	Bleomycin	I.V	OR: 45% (CR:5%) Median OS: 9.1 months	This study enrolled the HNC patients who had chemo and radio-refractory disease, having experienced at least two chemotherapy lines in addition to radiation therapy.	[89]
Pichi B et al.	2019	Prospective	36	SCC: 31 MM: 2 ADC: 1 Sarcoma: 1 Other: 1	Bleomycin	I.V	OR: 100% (CR:8.3%) 1-year OS: 41.6% Median OS: 9 months	This study focused on patients with heavily pre-treated recurrent mucosal and cutaneous HNCs.	[90]
Plaschke CC et al.	2019	Phase II	26	SCC: 25 ACC: 1	Bleomycin	I.V	OR: 58% (CR:19%)	This study focused on recurrent mucosal HNCs.	[91]
Jamsek C et al.	2020	Prospective	28	BCC: 42 lesions SCC: 10 lesions (Standard dose: 16 patients] BCC: 25 lesions SCC: 3 lesions (Reduced dose: 12 patients) BCC: 17 lesions SCC: 7 lesions	Bleomycin	I.V (standard dose):16 I.V (reduced dose):12	OR: Standard dose: CR:100% Reduced dose: CR:96% Tumor recurrence rate Standard dose (median follow-up of 28 months): 15.4% Reduced dose (median follow-up of 40 months): 39.0%	Patients with primary recurrent skin cancer of the head and neck aged > 65 years old were enrolled. This study compared standard I.V dose bleomycin (15,000IU/m^2^) and reduced doses (10.000IU/m^2^)	[92]
De Giorgi V et al.	2020	Prospective	8	BCC: 4 SCC: 3 Unknown: 1	Cisplatin	I.T	OR: 100% (CR:50%)	This study focused on skin cancer of the head and neck, except for cutaneous metastasis from breast cancer. Five patients were treatment-naïve	[48]

Abbreviations, N, number of patients; SCC, squamous cell carcinoma; ACC, adenoid cystic carcinoma; ADC, adenocarcinoma; MM, malignant melanoma; BCC, basal cell carcinoma; I.T, intratumoral injection; I.V, intravenous injection; OR, overall response; CR, complete response; LPFS, local progression-free survival; DFS, disease-free survival; PFS, progression-free survival; OS, overall survival. * The number indicates the number of patients who were treated with either I.V., I.T. or I.V. + I.T.
